# Is the university an authoritarian institution? A theoretical exploration with Lacan, Fromm, and Rancière

**DOI:** 10.3389/frma.2025.1579627

**Published:** 2025-07-18

**Authors:** Bastian Ronge

**Affiliations:** Institut für Philosophie, Bergische Universität Wuppertal, Wuppertal, Germany

**Keywords:** authoritarianism, university, Lacan, Rancière, Fromm, diversity education

## Abstract

This article examines the ambivalent role of the contemporary university in the face of rising authoritarianism. While universities are commonly perceived as bastions of humanism, committed to ideals such as freedom, critical inquiry, and Bildung, this optimistic view may obscure the fact that authoritarian dynamics can persist within the institution itself. Rather than labeling the university as an inherently authoritarian institution, the article argues that it constitutes a social field in which authoritarian tendencies may emerge and take effect—often in subtle and structurally embedded forms. The first part of the article reconstructs Jacques Lacan's psychoanalytic account of the university's authoritative structure and its transformation after 1968, focusing on how authority functions discursively within academic contexts. The second part draws on Erich Fromm's theory of the authoritarian character and combines his perspective with Lacan's framework to propose a heuristic model for identifying and analyzing authoritarian dynamics within present-day academic life. In the third part, the article turns to Jacques Rancière's concept of the ignorant schoolmaster as a means of outlining an anti-authoritarian pedagogy. Rancière's approach is presented as a practical and conceptual tool to carve out spaces of resistance and autonomy within the university. Finally, the article reflects on the limitations of its approach and suggests directions for future research.

“[W]riting critically about the academy is almost impossible.”

-David Graeber

## Introduction

Certainly, we live in times in which authoritarianism is on the rise. On first glance, the institution of university seems to be a stronghold against this trend. Built on the tradition of humanism, inhabited by subjects who are committed to norms like freedom, progress and *Bildung*, universities appear to be the antidotal to authoritarianism. This hopeful impression might be deceptive, however, given that the university itself entails authoritarian dynamics. At least, this is the claim, I would like to argue for in the following article. While I do not assert that the university *is* an authoritative institution, I maintain that the social field called university entails different authoritarian dynamics, which must be carefully analyzed if the university is to serve as a stronghold against the authoritarian tendencies of the present. I will start with a brief reconstruction of Jacques Lacan's psychoanalytical analysis of the authoritative structure of the university and its transformation after 1968 (Part 1). I then turn to Erich Fromm and his theory of the authoritarian character and attempt to combine in a rather speculative way the two theoretical engagements in order to develop an heuristic schema, which allows to describe and to analyze the potential authoritative dynamics within contemporary university (Part 2). Before this background I will discuss (briefly) the question of anti-authoritarian pedagogy by turning to Jacques Rancière and his concept of the ignorant schoolmaster. I will show that Rancière provides us with an anti-authoritarian pedagogy, which can help to create anti-authoritarian spaces within the present-day university (Part 3). In the very end, I discuss the limitations of my elaborations and hint at possible avenues for future research (Part 4).

## Lacan and the authoritarian discourse of the university

In the aftermath of the student revolt of 1968, Lacan develops his theory of the “four discourses,” mainly developed in his seminar on “the other side of psychoanalyses” (cf. Lacan, 2007). It allows him, amongst other, to analyze the political situation of his time and the students' demands of a “critical university” (Lacan, 2007). In this context Lacan postulates his well-known prophecy that the students' desire for overcoming bourgeois society and the traditional university will give birth to a new form of authoritarianism. “What you aspire to as revolutionaries is a master. You will get one” (Lacan, 2007, p. 207).^1^ What does Lacan mean with this enigmatic message? And has his prophecy become true? In discussing theses question, I will follow Henrik Bjerre, who elaborates in his essay “The Discourse of the University” not only the key concepts of Lacan, but also applies them to the university (cf. Bjerre, 2015).

According to Bjerre the *discourse of the master* is the most import of the four discourses and shows, how the “primordial [] structures of authority tend to function” (Bjerre, 2015, p. 46). Lacan uses the following “mathem” to illustrate the general logic of this discourse:


$$\begin{array}{l} \frac{{\text{S}}_{\text{1}}}{\$}\to \frac{{\text{S}}_{\text{2}}}{a} \end{array}$$

In Bjerre's interpretation the left upper space represents the agent of the discourse. In the case of the “discourse of the master” this position is hold by the master, which Lacan addresses as S1. The master acts upon an addressee, which is “knowledge” (S2) in this case, produced by the “master's definition and exclamation” (Bjerre, 2015, p. 46). The space below S2 is reserved for the product of the discourse, which is filled by the “knowledge worker” (Bjerre, 2015, p. 47) and his enjoyment of having knowledge (a). The space under S1 represents the truth of the whole discourse, which is in case of the discourse of the master the “barred subject” ($), i.e., the individual as subjected to the signification processes, which are initiated and authorized by the master.

This “basic type of social bond,” from which “all other discourses [are] being deducible” (Dolar, 2017, p. 139), can be used to analyse the traditional, pre-1968 university, which is dominated by the professor, which was—at these times—normally white and male. Bjerre writes:

“In the master's discourse, the master speaks and elicits commands. It is the master that informs knowledge, i.e., speaks the decisions that anchor discourse in certain unquestionable truism (master signifiers). We know this figure, of course, most intuitively from the character of the traditional, authoritarian father who corroborates his statements by a “because I said so!” Ultimately, there is nothing else that the father's enunciation to back his orders. It is so, because *he* says so. Priests and prophets speak in the master's discourse, and I would claim that it has always been spoken rather fluently in the academic world as well. Simplifying a little bit […] one could say that it was the traditional, male, white professor that students were rebelling against in 1968, and thus to a significant degree the master's discourse. It was the old, white men that defined academic standards and curricular through their very status as…old white men of academia.” (Bjerre, 2015, p. 47)

Lacan's prophecy that the revolutionary desire of the students in 1968 will bring about a new master can therefore be understood as a statement within the discourse of the master. Lacan speaks as a white, male professor and with the authority of S1, predicting the end of this kind of authority, since the new master will be the so-called “discourse of the university” (cf. Lacan, 2007).

It is easy to get confused here. Despite its name the discourse of the university does not originate in the university. On the contrary: The pre-1968 university is a paradigmatic case for the discourse of the master, since all of its social relationsships are determined by the ‘patriarchal' authority of the professor. The paradigmatic case for the “discourse of the university” is, therefore, not the university, but the Soviet Union as Lacan somewhat surprisingly states.

“The configuration of workers-peasants has nevertheless led to a form of society in which it is precisely the university that occupies the driving seat. For what reigns in what is commonly called the Soviet Union of Socialist republics is the university.” (Lacan, 2007, p. 206)

Thus, the word university does not refer to the institution university (cf. Gurschler, 2013, p. 107). Moreover, it refers to a kind of discourse, in which knowledge (S2) has been installed as the agent of the discourse, i.e., as the last authoritative instance of the social scenery.

The “mathem” of the discourse of the university can be deduced from the discourse of the master, “since it's just a matter of their circular permutation with the terms remaining in the same order” (Lacan, 2007, p. 43). So it looks like this:


$$\begin{array}{l} \frac{{\text{S}}_{\text{2}}}{{\text{S}}_{\text{1}}}\to \frac{a}{\$} \end{array}$$

Knowledge (S2) or, to be more precise, “nothing other than knowledge” (Lacan, 2007, p. 31) is now in the position of the agent and acts upon the knowledge worker (a) as the new addressee of the discourse. The product of this interaction is the subject ($), while the truth of the whole discourse is now embodied by the master (S1).

The discourse of the university starts to colonize the institution of the university as Bjerre argues with reference to the “changing role of the academic,” who understands his- or herself more and more as a “mere tool of knowledge.” Bjerre writes:

“The university discourse is a response to the master's discourse in this sense: it does not accept knowledge on the basis of authoritarian acts of enunciation. Knowledge does not depend on masters: instead, it speaks for itself. In the university discourse, knowledge stands in the place of the agent, the one issuing commands. The changing role of the academic can be seen in this light: more and more, academic researchers have declined from presenting knowledge on the basis of their authority […]. Instead, the researcher is presenting him or herself as a mere tool of knowledge—knowledge speaks through men, and its veracity depends not on my authority, but on its own emergence as facts, reports or observations.” (Bjerre, 2015, p. 48)

The traditional personal authority of the (white, male) professor is colonized by the anonymous authority of knowledge and knowledge production. This shows up in all the practices and procedures, which are typical for the neo-liberal university: Peer-review processes, metric analyses, ongoing evaluation processes etc. What has been called the neoliberal transformation of the university seems to correspond quite well, therefore, with Lacan's prophecy and his ascription of the “discourse of the university.”^2^ It would be a mistake, however, to understand the neoliberal transformation of the university as a complete replacement of the old, traditional structure of the university. Moreover, the neoliberal transformation leads to a condition, which the German sociologist Richard Münch has described in another context as hybrid modernization: New governmental techniques are amalgamized with the old ones (cf. Münch, 2009).

Contemporary universities are, therefore, spaces in which both discursive logics can be observed: the logic of the Master's discourse and that of the university discourse. At times, these logics appear in relatively “pure” form—for instance, in the senior professor who embodies the Master's discourse without deviation, or in the administration, which operates strictly within the logic of the university discourse. More often, however, both logics intersect and manifest within the same person or phenomenon.

The different ways, in which lectures can be delivered nowadays, is a good example to illustrate this coexistence of both logics. Bjerre writes:

“The technology of the university discourse is the power point presentation with informational slides and bullet points, whereas the technology of the master, one could say, is simply the voice. In the former, you get a thorough input of well-corroborated facts and information, and you might for instance merge your own notes with those of the lecturer, asking the teacher to provide you with secondary literature, discuss the uses in groups of fellow students, make you own presentations etc., while in the latter, you simply get the lecture—and are free to search for literature yourself.” (Bjerre, 2015, p. 49)

Lacan's prophecy is therefore right and wrong at the same time. Doubtlessly, the new public management of the university has installed the “discourse of the university” as a new authoritative structure within the university. At the same time, the “discourse of the master” is still alive. Hence, present-day universities should be described and analyzed as a social field, in which two different logics work with and against each other. With this “hybridity” in mind, I would like to turn to Erich Fromm and his theory of the authoritarian character. I am fully aware of the fact that Lacan and Fromm does not share or work on same theoretical grounds. Hence, the following is not meant as the attempt to complete Lacan's theory of the four discourses by Fromm's analyses of the authoritarian character. Moreover, I would like to complicate the heuristic schema, which can be developed with reference to Fromm, by Lacan's analyses of the university.

## Erich Fromm's theory of the authoritarian character

Erich Fromm's social psychology was a main pillar for the interdisciplinary research of the Institute for Social Research in its formative years.^3^ Especially his concept of the “authoritarian character”, developed within the empirical study on “Arbeiter und Angestellte am Vorabend des Dritten Reiches” in 1928,^4^ became significant to explain the missed revolution of 1918 and the rise of the Nazi fascism in Germany. According to Fromm, the bourgeois society generates various social character (Gesellschaftscharakter), depending on the social position of the subject.^5^ The authoritarian character, which is attracted to authoritarian solutions of social problems, has its social site in the bourgeoise family, with “the father” as personified figuration of authority. The authoritative subject develops an affective habitus which Fromm describes as a particular combination of two emotions which are seldom regarded as dialectical twins, namely the emotion of *admiration* and *contempt*.^6^

Whenever the authoritarian character is near “the authority,” he automatically feels love and admiration. Fromm writes:

“When this character type senses power, he almost automatically feels reverence and love. Thus, it matters little whether it is the power of a person, an institution or socially sanctioned idea. One could justifiably turn around the familiar proverb for him and say: ≫He loves the one who does not spare the rod≪ […]. This wish to receive orders and act upon them, to submit obediently to a higher power, or even to lose oneself in it completely, can go so far that he even enjoys being disciplined and mistreated.” (Fromm, 1936a/2020, p. 41)

The authoritarian character does not only feel joy in the wake of his subordination, but also hate and anger toward the authority, since he is responsible for his loss of freedom and autonomy. Since the authoritarian character does not allow himself to express these aggressive feelings toward the admired authority itself, he looks for alternative “receivers” for his negative feelings. The admired authority takes advantage of this need by demonizing foreign authorities and accepting and excusing aggressive behavior against marginalized people.

“When he loves someone stronger and more powerful, it does not mean that he does not also envy and hate him. Yet, this hate is usually repressed. Often ambivalence gets expressed in a sort of splitting. Some in power are ascribed all of the good qualities and are loved, whereas others are ascribed all of the bad qualities and are hated. Examples of this are the hatred toward the gods of other religions, toward the leaders of foreign peoples […] toward financial capital in contrast to ‘productive capital' […].” (Fromm, 1936a/2020, p. 41f.)

By blaming foreign authorities for any misfortune of the authoritarian character, the admired authority strengthens his emotional bond with him and mobilizes his negative and aggressive feelings against political opponents, like foreign political parties (“The Democrats”) or institutions (“The EU”).

Since anger and hate can usually not get acted out against those demonized others, the admired authority points at marginalized social groups within the society and allows his follower (explicitly or implicitly) to act out their hate and resentment against members of those groups. The (masochistic) enjoyment of subordination under the admired authority corresponds, therefore, with the (sadistic) enjoyment of mistreating and abuse weak and powerless subjects like women, migrants or homeless people. Fromm writes:

“All of the hostility and aggression present, which cannot be expressed toward the more powerful person, finds its object in the weaker one. If hatred against the strong must be repressed, at least cruelty toward the weak can be enjoyed. If asserting one's own will against the strong must be renounced, at least enjoyment can be found in the feeling of power provided by unrestrained control over the weak; and this means more control than just forcing the weak to suffer!” (Fromm, 1936a/2020, p. 42)

Although Fromm mostly uses “hate” (Haß) and seldom “*contempt*” *(Verachtung)* to describe this negative emotion of the authoritarian character, I would like to suggest to use the latter concept instead, since it allows to enrich Fromm's analysis in a very important way. In contrast to hate, contempt can be *hot* (like hate) or *cold* (like ignorance).^7^ In the latter emotional state, the subject overlooks “the other” and is not affected by him, his actions or reactions.^8^ His cold contempt can turn hot, however. Then, the subject acts aggressively toward the object of his contempt just like in the case of *hate*. Using the notion of *contempt* (instead of hate) helps us to understand that the practice of “ignoring” someone is just the other side (or antecedent) of hating and mistreating him or her.

Fromm's schema of the *authoritarian character* could, therefore, be sketched out like this:

**Subject**

(authoritarian character) feels admiration for the authority

                                        feels contempt for   foreign authorities

                                        *(cold/hot)*                 the weak and powerless

I think it is rather promising to apply Fromm' schema onto the Lacanian analysis of contemporary university as governed by two distinct authoritative discouress.

Let's start with the students, who enter the social field of the university for the first time. They will experience a double interpellation in the Althusserian sense^9^: They will be “hailed” by representatives of the discourse of the master, i.e. by (white, male) professor (in the classroom or in the photo-gallery on the homepage or as statues in front of the building) as well as by instances of the discourse of the university which calls for their subordination to the norms of knowledge production (this call might stem from professors, fellow-students or manuals). No matter which authority the incoming student will start to admire, his or her subordination will lead to feelings of contempt, which will be directed against instance of “foreign authorities” as well as “the weak and the powerless” on campus. Fromm's schema of the authoritarian character can, therefore, be used to make the “authoritarian” subjectivation of the incoming student visible:

**Student**

feels admiration for “the professor” or

                                 “norms of knowledge production”

feels contempt for    foreign authorities

*(cold/hot)*                  the weak and powerless

But who are the foreign authorities? And who stands for the weak and powerless on campus? Let's start with the latter question which is more easier to answer, since there is theoretical and empirical research on forms of marginalization and non-belonging on campus.^10^ Given this empirical data it seems plausible to ascribe the position of the weak and powerless to those students and groups of students, who do not fit the image of the traditional student, who used to be male, bourgeois, white ad heterosexual. The more students differ from this matrix, the more likely they become the target of the downward contempt of their fellow-students, who has subordinated to the authority of the professor or the norm of mere knowledge production.

Let's turn to the question of foreign authorities, which is much harder to answer. As we have learned from Fromm, the admired authority will often make use of the negative feelings of the subordinated subject to demonize other authorities and allow their expression toward the weak and the powerless. Given that we have to admire authorities within the academic field, complex affective dynamics can be expected: Perhaps one observes how representatives of the “discourse of the master” attempts to demonize “the administration” as some kind of foreign authority who is responsible for all misfortunes in the life of the students. Perhaps one observes how they assign (implicitly) members of marginalized groups on campus as legitimate objects of downward contempt. On the other had it might be the case, that the representatives of the discourse of the university point to the professorship and their institutional power as the main hindrance for realizing the full potential of the university as a scientific, knowledge producing institution, while treating non-traditional students as “second class” students, which have not the competences to act in accordance with the norms of knowledge production.

In light of this, in some parts rather speculative analysis, the present-day university appears as a social field, which is structured by complex authoritarian dynamics. The students, who enter the field, are subjectivized in a way, which does not immunize them against the call of authoritarianism which grasps more and more ground in contemporary politics. The hope that universities work as a natural stronghold against the rise of authoritarianism seems therefore to optimistic. If the university should become an institution which counteracts the rise of authoritarianism (in the Western hemisphere), this fight has to start within the walls of the university itself. In the last part I would like to turn to one important battleground, namely the realm of academic teaching.

## Stop admiring! The anti-authoritarian pedagogy of Jacques Rancière

Anarchism has experienced in recent years “a veritably renaissance” (Graeber, 2009, p. 105). This holds true not only for anarchism “[a]s a political philosophy” (Graeber, 2009, p. 105), as Graeber states, but also with regard to anarchism as a resource for “critical pedagogy”.^11^ This is hardly surprising, given that the critique of educational practices and institutions “has always been an integral component to anarchist theory” (Deleon and Love, 2009, p. 159).^12^

Although anarchists have a “long harbored skepticism toward formal academic institutions, which they tended to regards, highly, as ancillaries of the existing social, political, and economic order” (Jun, 2012, p. 283), there is also growing consent that it is possible to create anti-authoritarian spaces within the university by utilizing “its space, resources, skills, and knowledge's” in the right way to create “nomadic education machine[s]“ (Shukaitis, 2009, p. 167). But what is the right way? The analyses given above might be helpful to determine the general direction any answer to this complicated question must take. If the analysis was right, it has shown that the interpellation for admiration lies at the very ground of any authoritarian dynamics within the university. Any anti-authoritarian strategy has, therefore, to interrupt and to deconstruct the feeling of admiration, which is invoked in students in multiple ways. Or to put it more bluntly: *Stop Admiring!* should be the maxim of all anti-authoritarian strategies within the present-day university.

This would mean, however, a lot of work for anti-authoritarian practitioners. Since there are two admirable authorities—the professor and the norm of mere knowledge production—the interpellation for admiration is almost everywhere: It is incorporated in statues, portray galleries, homepages, How to…-scripts, training courses on scientific writing, and last but not least, in the behavior and the language of (almost all) members of the form of life called university. But the anti-authoritarian work is not only extensive, but also rather complex, since it could happen rather easily that the critique of one authoritative discourse unintentionally strengthens the other. It might seem rather promising, for example, to criticizes the discourse of the master by referring to the norms of mere knowledge production (like anonymous peer-revied processes or the praise of pure arguments etc.). But this critique strengthens the authority of the discourse of the university. The same dialectics play out if we attempt to criticize the latter by praising the genius and bookishness of the professor. In other words: Anti-authoritarian practice within the present-day university has to find ways of critique which deconstructs the authority of “the professor” *and* “the norms of knowledge” altogether without referring to one of them.

But how could we translate “this necessarily vague conceptualization into action” (Armaline, 2009, p. 142), i.e., into concrete pedagogical practices within the university? I am not able to discuss this question at length in this paper. What I would like to do, however, is to turn to one of the most controversially discussed books on teaching in the last years, namely to Jacques Rancière's study on “The Ignorant Schoolamster.”^13^

In his book Rancière criticizes all pedagogical settings, where the teacher is placed in the position of the knowing schoolmaster, who instructs its ignorant students by giving them explanations. According to Rancière, this traditional kind of teaching can never emancipate the student, since they will experience themselves as having an “inferior intelligence” in comparison to the “higher intelligence” of the teacher who remains always in the dominant position, since he or she “is the sole judge of the pint when the explication is itself explicated” (Rancière, 1991, p. 4). If pedagogy should have emancipatory effects, the “explicative order” (Rancière, 1991, p. 4) has to be left behind. But how could the teacher relate to his/her students without adopting the role of the knowing schoolmaster, who explains the students what they should learn?

The answer sounds rather simple, however disturbing. The teacher should not know what he or she teaches the students. He or she must be an ignorant schoolmaster—just like Joseph Jacotot, “a French schoolteacher who during his exile in Belgium in the first decades of the 19th century developed an education approach, which he called ‘universal teaching”' (Biesta, 2017, p. 60). Facing the challenge “to teach French to Flemish students whose language he didn't speak” (Biesta, 2017, 60), that is to say, without being able to explain anything to his students, Jacotot came up with the idea to provide them “a bilingual edition of Fénelon's novel *Télémaque*” (Biesta, 2017, p. 60) and forced them to compare the original text with the translation. To his own surprise, the method worked out. In the end the students were able to write French without making mistakes. They learned a foreign language without any explanatory discourse.

What seems utmost surprising at first sight, turns out to be quite normal, if one takes into account, that the students use the same method, which any child uses, when it learns its mother tongue.

“The child who repeats the words he hears and the Flemish student “lost” in his *Télémaque* are not proceeding hit or miss. All their effort, all their exploration, is strained toward this: someone has addressed words to them and they want to recognize and respond to, not as students or as learned men, but as people; in the way you respond to someone speaking to you and not to someone examining you: under the sign of equality.” (Rancière, 1991, p. 11)^14^

This “method of the riddle” which, according to Rancière, is “the true movement of human intelligence” (Rancière, 1991, p. 10), operates without any explanation. Teaching like the ignorant schoolmaster annihilates, therefore, the asymmetry which is constitutive for the relationship between the knowing schoolmaster and the unknowing student. The ignorant schoolmaster might have more knowledge than his students, but this knowledge is irrelevant, since his charge is not to transmit any knowledge to his students, but to foster their will to understand and to create opportunities in which this will is combined with the learning method of riddling, i.e., “by observing and retaining, repeating and verifying, by relating what they were trying to know to what they already knew, by doing and reflecting about what they had done” (Rancière, 1991, p. 10).

In this case, the students are not called to admire—neither the schoolmaster, who does not know anything more than the student, nor any norms of knowledge production, since the process of riddling cannot be described in terms of following a set of particular rules or norms. Hence, Rancière's thing-centered pedagogy” (Vlieghe, 2018) seems to fulfill the criterion, which we have formulated for any anti-authoritarian strategies within present-day universities. It allows to perform academic teaching without interpellating the students for admiration. The “teaching” of the ignorant schoolmaster is not about admiration, but about intense and continuous attention to the materiality of the thing which we want to understand. It is this attention that brings the teacher and the student together, as equal sentient beings, invested in the same adventure of understanding.

## Limitations and research perspectives

In conclusion, I would like to address the limitations of this article and outline some possible directions for future research. This article does not claim to provide a comprehensive discussion of the theories of Lacan, Fromm, or Rancière. Nor does it assert that the portrayal of the university as an authoritarian institution is empirically accurate. Rather, it aims to offer a heuristic framework that can be used to observe social interactions within the academic field and to foster critical self-reflection on the ways in which the university may (re)produce authoritarian affective dynamics. Such a critical reassessment appears particularly necessary in times of rising authoritarianism.

Against this backdrop, several avenues for future research suggest themselves. It would be both interesting and important to test the heuristic framework developed here using empirical methods. Furthermore, the discussion of abolition could be brought more explicitly into the picture. Emerging from the context of decolonial critique (cf. Rodriguez, 2012), abolition may offer a radical approach to dismantling the admirable aura that surrounds university campuses. Finally, it seems promising to integrate Lacan's and Fromm's theories about authority and the authoritarian character into touch with recent anarchist critiques of contemporary academia. Although, “the rejection of authority is the very basis of anarchism” (Newmann, 2001, p. 158), the analyses offered by Lacan and Fromm plays, as far as I see, no prominent role in these discussions. Last, but not least it would be crucial to investigate the mechanisms and conditions, under which cold contempt transforms into an hot emotion; particular in an age of rising authoritarianism.

## Footnotes

^1^For this prophecy and its historical context: Copjec (2006).

^2^For a more detailed description of this neoliberal transformation, (cf. Readings, 1996; Hark and Hofbauer, 2018, 2023).

^3^Cf. Bonß (1992, p. 23).

^4^For more details about this study and the important role of Hilda Weiß for its realization, (cf. Bonß, 1980, p. 1f; Smith, 2020; Steinhauer, 2023, p. 113–116).

^5^For a critique of this thesis (cf. Dahmer, 1973).

^6^That the emotions of admiration and contempt relates to each other is most clearly acknowledged by Spinoza in his Ethics.

^7^This distinction between hot and cold contempt is adopted from Burkhard Liebsch (cf. Liebsch, 2007).

^8^Again it is Spinoza who acknowledges this ignorant aspect of contempt (cf. Spinoza, 2020, Part 3 Def 5).

^9^The *interpellation* which Althusser illustrates by the example of being called by a police officer, describes a mode of being subjectivized which is “opposed to ‘recognition,”' since it “implies an inequality of force” (Montag, 2013, p. 137). Hence, acts of interpellation install a social relationship which is constitutively asymmetrical, while acts of recognition establish symmetrical social relationships.

^10^Cf. Rieger-Ladich et al. (2020). With regard to racial campus climate and its effects on non-white students (cf. for example Reid and Radhakrishnan, 2003; Hoffman, 2018).

^11^For the field of critical pedagogy (cf. Kincheloe, 2004; McLaren, 1994).

^12^For this complex (cf. Amster et al., 2009).

^13^Reading Rancière as an anarchist is quite productive as Anna-Teresa Steffner shows (cf. Steffner, 2017).

^14^Rancière's description is strongly reminiscent of Jean Laplanche's elaborations on the enigmatic messages in the context of his theory of seduction (cf. Laplanche, 1999). This connection might be interesting for further elaboration.
